# Long-Term Progressive Degradation of the Biological Capability of Titanium

**DOI:** 10.3390/ma9020102

**Published:** 2016-02-06

**Authors:** Hajime Minamikawa, Wael Att, Takayuki Ikeda, Makoto Hirota, Takahiro Ogawa

**Affiliations:** 1 Department of Molecular Cell Pharmacology, Graduate School of Dental Medicine, Hokkaido University, Sapporo 060-8586, Japan; 2 Laboratory for Bone and Implant Sciences (LBIS), The Weintraub Center for Reconstructive Biotechnology, Division of Advanced Prosthodontics, UCLA School of Dentistry, Los Angeles, CA 90095-1668, USA; wael.att@uniklinik-freiburg.de (W.A.); ikeda.takayuki@nihon-u.ac.jp (T.I.); hirotamakoto@me.com (M.H.); togawa@dentistry.ucla.edu (T.O.); 3 Department of Prosthodontics, Dental School, Albert-Ludwigs University, Freiburg 79106, Germany

**Keywords:** bone-implant integration, osseointegration, biological aging, dental and orthopedic implants, hydrophilicity

## Abstract

Titanium undergoes time-dependent degradation in biological capability, or “biological aging”. It is unknown whether the biological aging of titanium occurs beyond four weeks and whether age-related changes are definitely associated with surface hydrophilicity. We therefore measured multiple biological parameters of bone marrow-derived osteoblasts cultured on newly prepared, one-month-old, three-month-old, and six-month-old acid-etched titanium surfaces, as well as the hydrophilicity of these surfaces. New surfaces were superhydrophilic with a contact angle of ddH_2_O of 0°, whereas old surfaces were all hydrophobic with the contact angle of around 90°. Cell attachment, cell spread, cell density, and alkaline phosphatase activity were highest on new surfaces and decreased in a time-dependent manner. These decreases persisted and remained significant for most of the biological parameters up to six-months. While the number of attached cells was negatively correlated with hydrophilicity, the other measured parameters were not. The biological capability of titanium continues to degrade up to six months of aging, but these effects are not directly associated with time-dependent reductions in hydrophilicity. A full understanding of the biological aging will help guide regulatory improvements in implant device manufacturing and develop countermeasures against this phenomenon in order to improve clinical outcomes.

## 1. Introduction

The recent discovery that titanium undergoes time-dependent degradation, or biological aging, has provided new insights in biomaterial research with significant potential for therapeutic impact in the field of implant therapy and reconstructive medicine [1,2,3,4,5,6,7]. Biological capability, such as the number of attached osteogenic cells, proliferative activity, and functional phenotype of the cells, are significantly reduced on old titanium surfaces compared to new titanium surfaces [3,8,9]. For instance, the number of osteoblasts attached to four-week-old titanium surfaces is reduced by 30%–70% compared to new surfaces [3,8]. Accordingly, the osteoconductivity of four-week-old titanium, evaluated by the strength of bone-titanium integration and the areas of bone-titanium contact, is reduced to less than half of new titanium [3,8]. Time-dependent degradation occurs on various titanium surfaces, including machined, sandblasted, and acid-etched surfaces, and on deposited titanium [2,3,8,10].

Although the exact mechanism of biological aging of titanium is unknown, the reduced biological capability is associated with time-dependent reduction in hydrophilicity [2,3,8,9,10,11]. New titanium surfaces are hydrophilic, whereas sufficiently aged titanium surfaces are hydrophobic. Since the titanium surfaces used for experimental and therapeutic purposes are aged, they are most likely hydrophobic [5,6,7,12]. It is also known that four-week-old titanium surfaces are already hydrophobic, since the contact angle of water is greater than 60° [1]. However, the exact contribution of the degree of hydrophilicity in determining the biological capability of titanium is still contentious, and indeed there is no significant correlation between the degree of hydrophilicity and protein adsorption or the number of attached cells [1].

Another time-sensitive property of titanium is the atomic percentage of surface carbon [1]. Carbon molecules, in the form of hydrocarbon, unavoidably accumulate on titanium surface over time [1]. The percentage of surface carbon increases from less than 20%, to higher than 60%, correlated with the age of titanium [1]. Conversely, there is a significant negative correlation between the amount of surface carbon and cell attachment, suggesting that carbon contamination is a critical determinant of the biological capability of titanium [1]. Commercial implant products are significantly contaminated with carbon-containing molecules [5,6,13,14], suggesting that this factor is likely to be clinically significant.

There are still important questions to be answered regarding the biological aging of titanium. Previous studies have only examined the aging process up to four weeks, and it is unknown whether the degradation of biological capability continues after this time and for how long. Given that the current cycle of manufacturing, distribution, and sales of implant and other titanium-based materials rarely occurs within one month, understanding these properties is of extreme importance to both the manufacturing and clinical communities. The exact role and impact of reductions in hydrophilicity on the biological aging of titanium also needs to be addressed. Since the change in hydrophilicity on titanium over the long term has not been investigated, there is currently no information available to speculate on its biological role. We therefore measured the biological capability of titanium during six months of aging, with a particular focus on its interaction with osteogenic cells. The response and behavior of osteogenic cells were examined with respect to the degree of hydrophilicity of titanium over time.

## 2. Results

### 2.1. Surface Morphology of Titanium Samples

**Figure 1:**
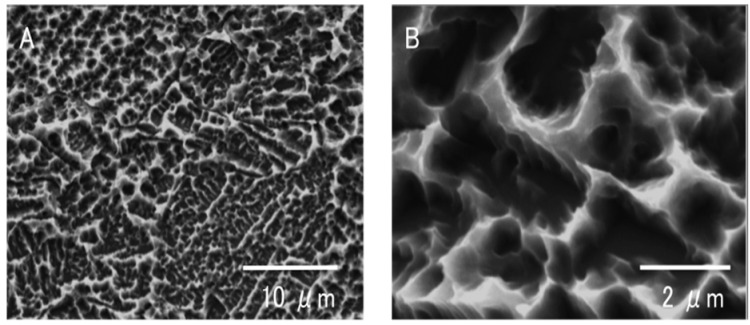

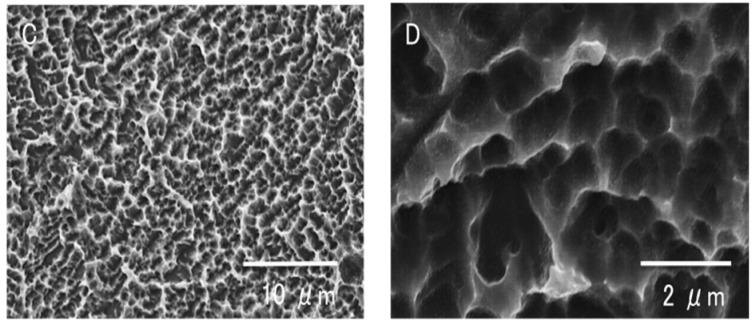
Scanning electron microscopic (SEM) images of new titanium samples (**A**,**B**) and 6-month-old titanium samples (**C**,**D**) in low and high magnifications.

Low magnification SEM image of new titanium surface showed uniform and even formation of surface roughness (Figure 1A). High magnification images of new titanium surfaces showed the typical microtopography formed by acid-etching, consisting of pits and sharp peaks with an interval of 0.5–2.5 μm (Figure 1B). The 6-month-old titanium surface showed very similar surface structure and there were no recognizable changes between new and 6-month-old surface in roughness, uniformity and appearance of the micropit features in low and high magnification images (Figure 1C,D).

### 2.2. Hydrophilicity Change During Titanium Aging

**Figure 2:**
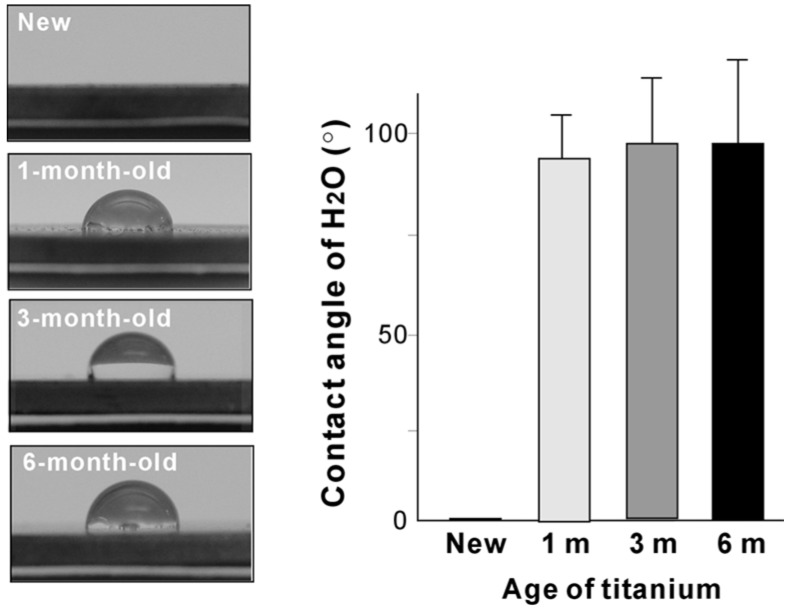
Hydrophilic or hydrophobic status of new and differently aged titanium surfaces. Side-view images of 10 μL·ddH_2_O placed on titanium disks along with the measured contact angle. The contact angle on all aged surfaces was significantly greater than that on new surface (*p* < 0.001), whereas there was no significant difference among the differently aged surfaces.

Photographic images of 10 μL ddH_2_O placed on the titanium disks are shown in Figure 2. Water droplets spread immediately over the entire area of new titanium surfaces. The contact angle of water was 0.0°, indicating that the new surfaces were superhydrophilic. In contrast, water droplets formed hemispheric droplets with a contact angle of approximately 90° on 1-month-old titanium surfaces, indicating that the surfaces were hydrophobic. Similarly, 3-month-old and 6-month-old surfaces were hydrophobic. Although the contact angle on the aged surfaces was significantly greater than that on the new surface, there were no significant differences between the three differently aged surfaces.

### 2.3. Number of Attached Cells

**Figure 3:**
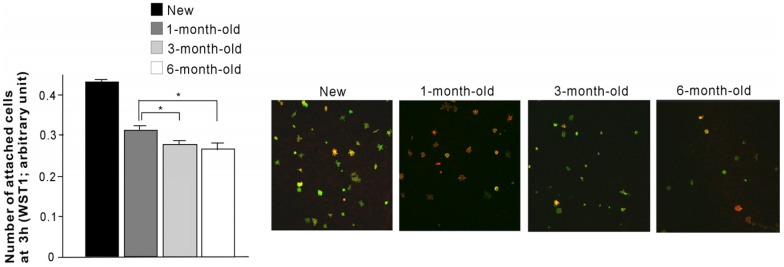
The number of osteoblasts attached to new and differently aged titanium surfaces during a 3-h incubation. Representative confocal microscopic images of osteoblast culture on each of the titanium surfaces are also presented. The number of attached cells on aged surfaces (1-month-old, 3-month-old, and 6-month-old surfaces) was all significantly lower than that on new surfaces (*p* < 0.01). * *p* < 0.05, statistically significant difference among differently aged surfaces.

There was a significant association between the age of titanium and the number of cells attaching during 3-h of incubation (*p* < 0.01; Figure 3). The number of attached cells was highest on the new surface, followed by 1-month-old, 3-month-old, and 6-month-old surfaces. The number of cells attached to old surfaces was significantly lower than the number attached to new surfaces regardless of how old they were (*p* < 0.01). The number of cells attached to 3-month-old and 6-month-old surfaces were fewer than the number attached to 1-month-old surfaces, whereas there was no significant difference between 3-month-old and 6-month-old surfaces. Low magnification images using confocal microscopy confirmed the results by showing that there were more cells attached on new surfaces than aged surfaces and that there was a decreasing trend with age.

### 2.4. Initial Behavior of Cells

**Figure 4:**
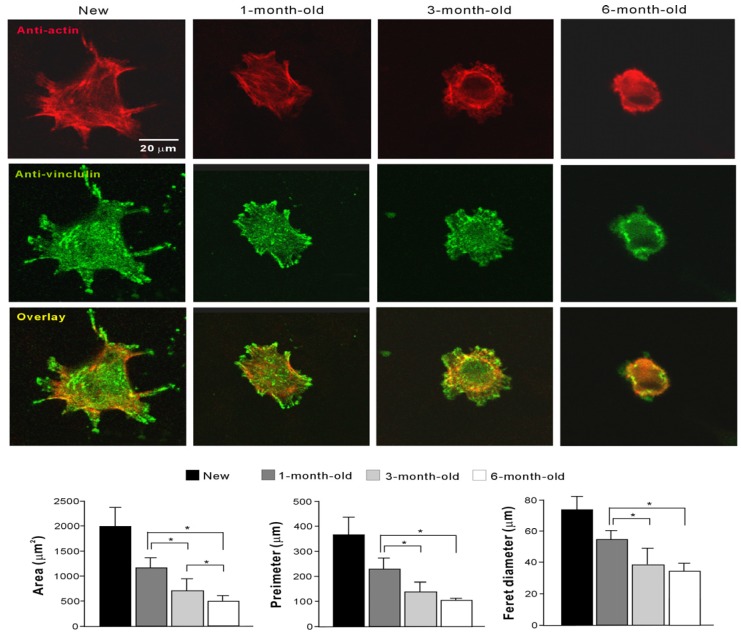
Attachment and spreading behavior of osteoblasts on new and differently aged titanium surfaces. Confocal microscopic images of osteoblast with immunochemical stain for cytoskeletal actin and adhesion protein, vinculin are shown. Cytomorphometric parameters measured from the images are presented at the bottom. All parameters were significantly lower on the aged surfaces (1-month-old, 3-month-old, and 6-month-old surfaces) than on new surfaces (*p* < 0.05). * *p* < 0.05, statistically significant difference among differently aged surfaces.

Magnified confocal images of cells after a 3-h incubation revealed that cells on new surfaces were larger than those on old surfaces (Figure 4). Cytoplasmic projections, such as filopodia and lamellipodia, had already developed in most cells on new surfaces even at this early stage of culture. Intense expression of vinculin was detected at the tip of projections. Moreover, a cytoplasmic actin cytoskeleton, as detected by rhodamine, was visible in cells cultured on new surfaces. In contrast, cells cultured on aged titanium surfaces were smaller and the cytoskeleton and cytoplasmic projections were either not at all visible or were only at the very earliest stages of development. All cytomorphometric parameters were significantly greater for the cells cultured on new surfaces than on old surfaces (histograms in Figure 4). Even among the old surfaces, there were significant decreasing trends in these parameters with increasing titanium age. There was a significant difference between 1-month-old and 3-month-old surfaces for all parameters, and there was significant difference between 3-month-old and 6-month-old surfaces in the cell area.

### 2.5. Number of Propagated Cells and Functional Phenotypes

**Figure 5:**
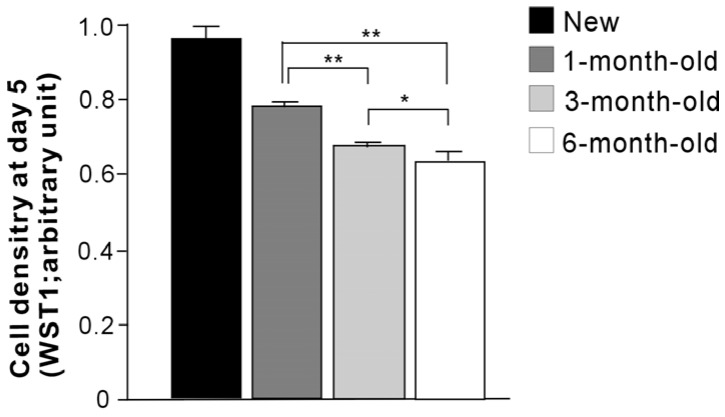
Cell density measured at day 5 of couture. The cell density was significantly lower on all aged surfaces (1-month-old, 3-month-old, and 6-month-old surfaces) than on new surfaces (*p* < 0.05). ** *p* < 0.01, * *p* < 0.05, statistically significant difference among differently aged surfaces.

**Figure 6:**
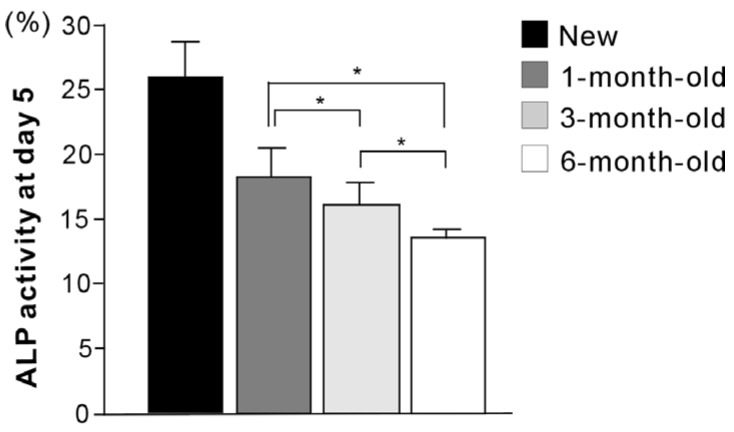
Alkaline phosphatase (ALP) activity at day 5 of culture on new and differently-aged titanium surfaces. The ALP activity was significantly lower on all aged surfaces (1-month-old, 3-month-old, and 6-month-old surfaces) than on new surfaces (*p* < 0.05). * *p* < 0.05, statistically significant difference among differently aged surfaces.

**Figure 7:**
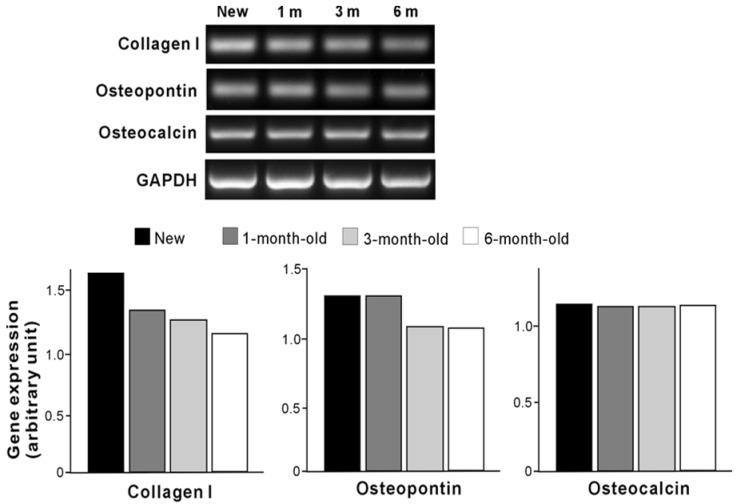
Expression of bone-related genes in osteoblast cultures at days 7 examined by reverse transcriptase-polymerase chain reaction (RT-PCR). Expression levels were quantified relative to the level of GAPDH mRNA expression.

The number of cells propagated during a mid-stage of culture was evaluated by assessing cell density at day 5 (Figure 5). There were fewer cells on all aged surfaces than on new surfaces. Significant decreases in cell density continued to occur with increasing titanium age up to 6 months. The ALP-positive area was evaluated as a marker of osteoblastic function (Figure 6), and was significantly smaller on aged surfaces. The progressive decrease remained significant between 3-month-old and 6-month old titanium. The expression of osteogenic genes was also evaluated at day 7 of culture (Figure 7). There was a trend of downregulation of collagen I and osteopontin gene expression with increasing titanium age. Osteocalcin expression remained unchanged.

### 2.6. Biological Parameters in Relation to Hydrophilicity

**Figure 8:**
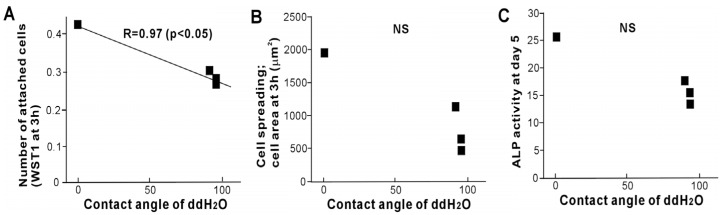
Plot of biological parameters against the contact angle of ddH_2_O. The number of attached cells at 3 h (**A**); cell area at 3 h (**B**) and ALP activity at day 5 (**C**) plotted in association with the contact angle of ddH_2_O. A significant inverse linear correlation was found only for the number of attached cells.

To assess the role of hydrophilicity in relation to the biological capability of titanium, potential correlations were examined between the degree of hydrophilicity on aging titanium and the three biological parameters (Figure 8A). The number of attached cells was negatively correlated with hydrophilicity with the coefficient of correlation being as high as 0.97, whereas the cell areas at 3 h and ALP-positive area were not correlated with hydrophilicity (Figure 8B,C).

## 3. Discussion

Here we demonstrate that the degradation of the biological capability of titanium is time-dependent and progresses up to six months. All the biological parameters tested in the study significantly decreased between 1-month-old surfaces and 6-month-old surfaces, and the functionally important parameters, such as the spread area, number of propagated cells, and ALP activity, decreased between 3 months and 6 months. These results indicate for the first time that there are significant reductions in the biological capability of titanium even after 1 month, further advancing our understanding of the biological aging of titanium.

The number of cells attaching during the very earliest stages of the cell-titanium interaction correlated with the degree of hydrophilicity, with more cells attaching to more hydrophilic titanium surfaces. However, the density of propagated cells and the spreading behavior did not correlate with hydrophilicity. While most of the biological parameters examined in this study decreased in a time-dependent manner on titanium surfaces older than 1 month, the level of hydrophobicity plateaued at 1 month, re-affirming the lack of correlation between them. These results suggest that the number of attached cells, which is predominantly dependent on the remote physicochemical interaction between the cells and titanium, is influenced by the hydrophilicity of titanium but that subsequent cellular behavior and function are either not at all, or less dependent, on hydrophilicity. In the general field of biomaterials, the role of substrate hydrophilicity in determining bioactivity is contentious [15], and it is not universally accepted that the more hydrophilic the surface, the more biocompatible the biomaterial [16,17,18,19,20]. For instance, a polymer surface with improved hydrophilicity has been shown to reduce fibroblast proliferation [18], and more hydrophobic polymer scaffold materials are effective in promoting bone regeneration [20]. On titanium surfaces, the number of attached cells does not necessarily correlate with the degree of hydrophilicity [1,11,21]. While there are commercially available hydrophilic dental implants, which are stored in specific solution [12,22], fewer cells attach to these hydrophilic surfaces than to hydrophobic surfaces with identical surface morphology [16]. Therefore, there are likely to be other parameters responsible for the observed effects of biological aging, such as the amount of surface carbon (*i.e.*, how chemically clean titanium surfaces are), which need further study.

By demonstrating these biological effects over the long-term aging of titanium, we provide novel insights into the biomedical science of titanium. To the best of our knowledge, the age of titanium surfaces has not been standardized or previously described in the literature on implant and titanium research. The continuous and substantial age-dependent changes of titanium bioactivity observed in this study may prompt more careful time-dependent monitoring of biological properties over longer time-frames. Future studies that compare different titanium surfaces for their biological and osteoconductive capabilities need to document the age of these samples to allow more precise and reasonable interpretation of results.

We confirm that the biological aging of titanium is an important and real phenomenon that is likely to contribute to therapy and clinical efficacy. Implant products, regardless of whether for dental or orthopedic use, are packed sterile and sold as storable medical devices at both the manufacturer and user level. It is therefore generally believed that the biological properties of implant surfaces remain stable over time, with the osseointegration capability of titanium assumed not to vary, regardless of when the surface was prepared. Until now, the bioactivity of implant materials over time has generally been ignored, and the implications of this on the shelf life of implant products remains unaddressed. For instance, the manufacturing date of implants is not available to users, the primary parameter for acceptable use being the expiration date of sterilization (generally five years); the instructions and product information fail to regard implant age. Here we demonstrate that biological aging is not just a rapid and instant phenomenon occurring within one month of surface preparation, but progressive and worsening over the longer term, at least up to six months. This has important clinical implications that require counter measures, and also implications from a statutory and regulatory perspective for device manufacturers.

In addition to revealing concerns about implant manufacturing and storage, these and previous data also improve our understanding of the process of osseointegration by revealing why the percentage of bone-implant contact does not reach an ideal 100%. We previously demonstrated that greater than 90% bone-implant contact can be obtained by using new titanium surfaces, compared to less than 60% for aged surfaces [1]. This suggests that the innate bioactivity of titanium implant products is much greater than that usually obtained from commercially available products that undergo aging over an unidentified time period [3,5]. This has two main implications for future improvement of implant surfaces; firstly to prevent biological aging and secondly to counteract aging. Hydrophilicity can be maintained by storing titanium in liquid, which is an effective preventative strategy [12,22]; however, there is little information regarding the effect of liquid-based storage on other physicochemical properties, such as carbon percentage, electrostatic status, and time-related changes, making interpretation of the effect of these liquid-stored surfaces difficult. With regards to counteracting aging, photofunctionalization is a novel approach with promising potential to overcome the biological aging of titanium [3,4,6,7,21]. Photofunctionalization is the physicochemical and biological change induced by treating titanium surfaces with specific UV light, and results in physicochemical reversal of the aging phenomenon by regenerating hydrophilicity, removing hydrocarbon, and optimizing the electrostatic properties of the surface [23,24].

## 4. Materials and Methods

### 4.1. Titanium Samples and Surface Characterization

Disks (20 mm in diameter and 1.5 mm in thickness) fabricated from commercially pure titanium were acid-etched with 67% H_2_SO_4_ at 120 °C for 75 s. The disks were placed in a sealed container and stored in a dark room (temperature 23 °C; humidity 60%) for 0 h (new), 4 weeks (1-month-old), 12 weeks (3-months-old), and 24 weeks (6-months-old). The surface morphology was examined using scanning electron microscopy (SEM) (XL30, Philips, Eindhoven, The Netherlands). The hydrophilic or hydrophobic status of titanium surfaces was evaluated by measuring the contact angle of 10 μL·ddH_2_O.

### 4.2. Osteoblast Cell Culture

As established previously [25], bone marrow-derived osteoblastic cells were isolated from the femurs of 8-week-old male Sprague–Dawley rats and placed into alpha-modified Eagle’s medium supplemented with 15% fetal bovine serum, 50 µg/mL ascorbic acid, 10 mM Na-β-glycerophosphate, 10^−8^ M dexamethasone, and antibiotic–antimycotic solution containing 10,000 units/mL penicillin G sodium, 10,000 mg/mL streptomycin sulfate, and 25 mg/mL amphotericin B. Cells were incubated in a humidified atmosphere of 95% air and 5% CO_2_ at 37 °C. At 80% confluency, the cells were detached using 0.25% trypsin-1 mM EDTA-4Na and seeded onto titanium disks placed in a 12-well culture dish at a density of 4 × 10^4^ cells/cm^2^. The culture medium was renewed every 3 days.

### 4.3. Cell Attachment and Density Assays

Initial attachment of cells was evaluated by measuring the number of cells attached to titanium disks after 3 h of incubation. Propagated cells were also quantified as cell density on day 5 of culture. These quantifications were performed using WST-1-based colorimetry (WST-1, Roche Applied Science, Mannheim, Germany). A culture well was incubated at 37 °C for 4 h with 100 µL of tetrazolium salt (WST-1) reagent. The amount of formazan product was measured using an ELISA reader at 420 nm (Synergy HT, BioTek Instruments, Winooski, VT, USA).

### 4.4. Morphology and Spreading Behavior of Osteoblasts

Spreading behavior and cytoskeletal arrangement of osteoblasts seeded onto titanium surfaces were examined using confocal laser scanning microscopy. Three hours after seeding, cells were fixed in 10% formalin and stained using rhodamine phalloidin, a fluorescent dye (actin filament, red color; Molecular Probes, Eugene, OR, USA). To observe the intracellular expression and localization of vinculin, a focal adhesion protein, cells were additionally stained with mouse anti-vinculin monoclonal antibody (Abcam, Cambridge, MA, USA), followed by FITC-conjugated anti-mouse secondary antibody (Abcam, Cambridge, MA, USA). The area, perimeter, and Feret’s diameter were quantified using an image analyzer (ImageJ, NIH, Bethesda, MD, USA).

### 4.5. Alkaline Phosphatase (ALP) Activity

ALP activity of osteoblasts was examined on day 5 using image-based densitometry. Cultured cells were washed twice with Hanks’ solution and then incubated with 120 mM Tris buffer (pH 8.4) containing 0.9 mM naphthol AS-MX phosphate and 1.8 mM fast red TR for 30 min at 37 °C. The ALP-positive area on the stained images was calculated as [(stained area/total dish area) × 100](%) using an image analyzer (ImageJ, NIH, Bethesda, MD, USA).

### 4.6. Gene Expression Analysis

Gene expression was analyzed by reverse transcription-polymerase chain reaction (RT-PCR) at day 7. Total RNA was extracted from the cells using TRIzol (Invitrogen, Carlsbad, CA, USA) on a purification column (RNeasy, Qiagen, Valencia, CA, USA). Following DNAse I treatment, reverse transcription of 0.5 μg of total RNA was performed using MMLV reverse transcriptase (Clontech, Carlsbad, CA, USA) in the presence of oligo (dT) primers (Clontech). PCR was performed using Taq DNA polymerase (EX Taq, Takara Bio, Madison, WN, USA) to detect type I collagen, osteopontin, and osteocalcin mRNA using the primer designs and PCR conditions optimized previously [25,26]. PCR products were visualized on 1.5% agarose gel with ethidium bromide staining. Band intensity was normalized to GAPDH mRNA.

### 4.7. Statistical Analyses

Hydrophilicity was evaluated on 3 different titanium disks (*n* = 3). Three disks were used for all cell culture studies (*n* = 3), except for the analysis of cytomorphometry where 6 independent cells were evaluated (*n* = 6). One-way ANOVA was performed to examine the difference between differently aged titanium groups; *p* < 0.05 was considered statistically significant. When needed, the post-hoc Bonferroni test was used as a multiple comparison. Correlations between biological parameters and the degree of hydrophilicity on titanium surfaces were determined using the least-mean-squares approximation.

## 5. Conclusions

The biological capability of titanium surfaces has not previously been studied over one month. We examined newly prepared, one-month-old, three-month-old, and six-month-old surfaces, and established that new surfaces are superhydrophilic, whereas all of the aged surfaces are hydrophobic. The number of attached cells, rate of cell spread, cell density, and ALP activity in osteoblasts were highest on new surfaces and decreased on old surfaces in an age-dependent manner. While the number of attached cells negatively correlated with the degree of hydrophilicity of titanium, the other biological parameters did not, denying a direct connection between hydrophilicity and biological capability of titanium and suggesting other factors may be responsible for the observed biological effects. The biological capability of titanium continues to degrade during aging up to six months, and is not necessarily associated with time-dependent disappearance of hydrophilicity.

## Abbreviations

The following abbreviations are used in this manuscript:

### Abbreviations

SEM: Scanning electron microscopy;
ALP: Alkaline phosphatase;
RT-PCR: Reverse transcription-polymerase chain reaction.
